# Genome of *Rhodovulum iodosum*, a marine photoferrotroph

**DOI:** 10.1128/mra.00607-24

**Published:** 2025-07-29

**Authors:** Giorgio Bianchini, Verena Nikeleit, Andreas Kappler, Casey Bryce, Patricia Sánchez-Baracaldo

**Affiliations:** 1 School of Geographical Sciences, University of Bristol 152335 , Bristol, United Kingdom; 2 Department of Geosciences, University of Tübingen 9188 , Tübingen, Germany; 3 Energy & Technology, NORCE Norwegian Research Centre 521537 , Bergen, Norway; 4 Cluster of Excellence: EXC 2124: Controlling Microbes to Fight Infections, University of Tübingen, Tübingen, Germany; 5 School of Earth Sciences, University of Bristol 152333 , Bristol, United Kingdom; University of Maryland School of Medicine, Baltimore, Maryland, USA

**Keywords:** photoautotrophy, photoferrotrophs, genomes

## Abstract

Photoferrotrophs drove primary production in the Earth’s early oceans and are still abundant in modern environments. We present a draft genome sequence of a model photoferrotroph, *Rhodovulum iodosum* UT/N1. By comparing it with its close relative *Rhodovulum robiginosum* DSM 12329, we identify that these cultures were likely swapped during their history.

## MATTERS ARISING

Anoxygenic phototrophic Fe(II) oxidizers, or photoferrotrophs, oxidize Fe(II) and harness sunlight for energy in order to drive fixation of CO_2_ into biomass. They are thought to have dominated Earth’s early marine environments, though in modern times they are restricted to distinct sunlit and anoxic niches. Nevertheless, they still play an important role in redox-stratified lakes and are present across a broad range of environments (1). Despite their abundance, only a small number of photoferrotrophs have been isolated, belonging to the green sulfur bacteria (GSB) and purple (non)-sulfur bacteria (PSB). The majority are derived from freshwater systems, with only three marine photoferrotrophs available in culture collections: the PSB *Rhodovulum iodosum* and *Rhodovulum robiginosum* and the GSB *Chlorobium* sp. N1 (2).

**Fig 1:**
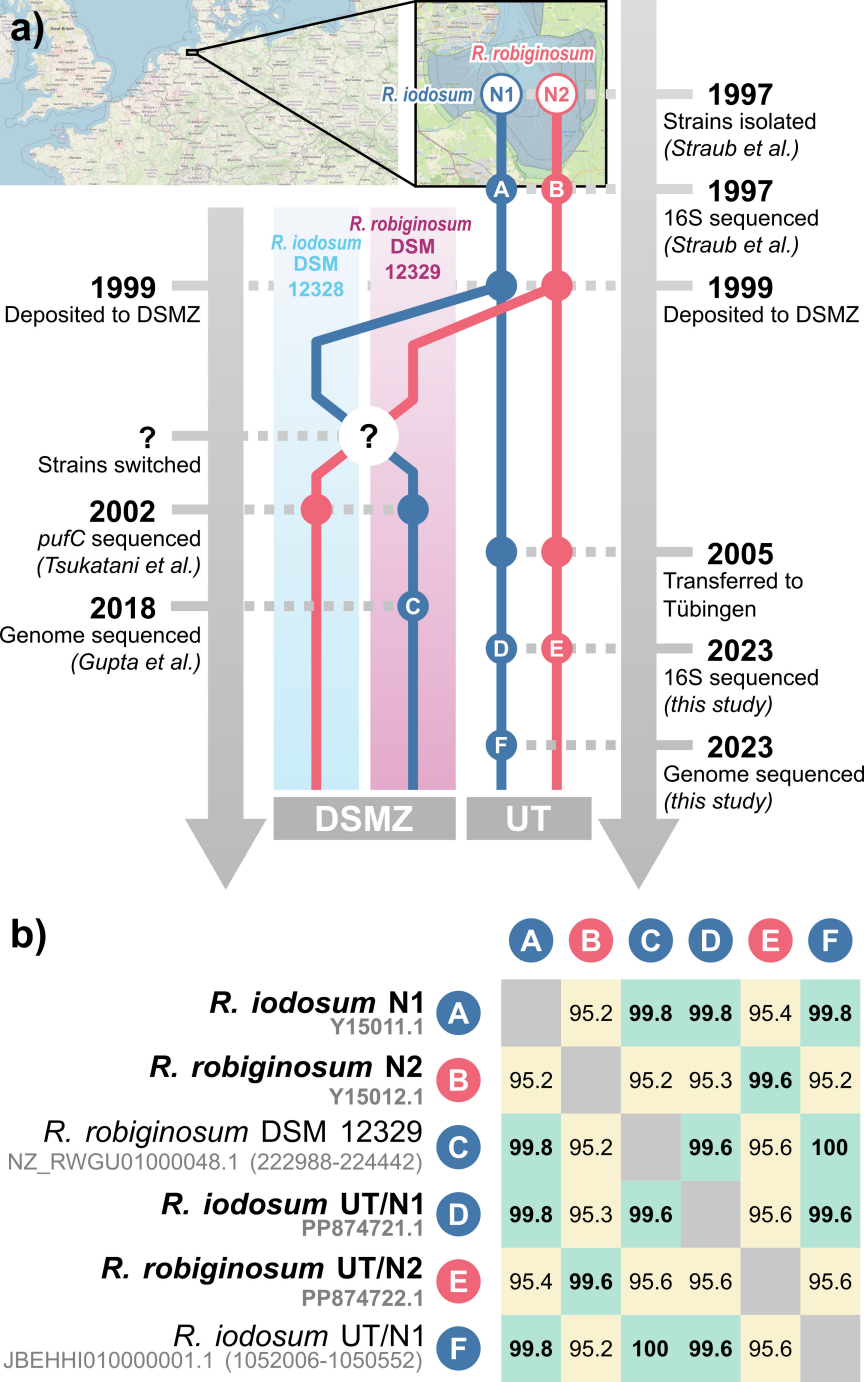
History of *Rhodovulum* cultures. (a) Two strains (*R. iodosum* N1 and *R. robiginosum* N2) were isolated from the Jadebusen (North Sea, Germany) in 1997, their 16S sequences were determined, and they were deposited in the DMSZ (3). Between 1999 and 2002, a switch likely occurred in the DSMZ. In 2002, *pufC* sequences were obtained (7), and in 2018, the *R. robiginosum* genome was sequenced (8), both from samples obtained from the DSMZ. In this study, 16S sequences were obtained for both *R. iodosum* and *R. robiginosum*, and the *R. iodosum* genome was sequenced, from samples held at the Eberhard Karls Universität Tübingen that directly descend from the original N1 and N2 strains. Letters within the timeline refer to 16S sequences analysed in part b. (b) Percent identity matrix between 16S sequences obtained from *R. iodosum* and *R. robiginosum* strains. Each sequence is identified by the name of the strain from which it was obtained and by its GenBank accession number. Circled letters refer to the timeline in part a. Sequences highlighted in bold (A, B, D, E) were obtained through Sanger sequencing, while the other sequences (C, F) were extracted from genome assemblies. Within the identity matrix, values higher than 99.5% are highlighted in bold and green, while values lower than 99.5% are highlighted in yellow. Map data from OpenStreetMap (https://openstreetmap.org/copyright).

Straub et al. (3) isolated the two marine PSB photoferrotrophs from the mud flat of the Jadebusen (North Sea, Germany) in 1997. The two strains, sharing 95.2% 16S ribosomal RNA (rRNA) identity, were described as *Rhodovulum iodosum* N1 and *Rhodovulum robiginosum* N2 and were deposited to the German Collection of Microorganisms and Cell Cultures (DSMZ; Braunschweig, Germany) as DSM 12328 and DSM 12329, respectively. Furthermore, samples from both strains were kept in continuous culture at the Eberhard Karls Universität Tübingen (UT, Germany) since approximately 2005 (Fig. 1a). These strains have been widely used as models in the study of marine photoferrotrophy (4–6). Here, we present a draft genome of the Tübingen culture of *R. iodosum* (which we will term *R. iodosum* UT/N1), which, until now, was the only marine photoferrotroph with no available genomic information.

*R. iodosum* UT/N1 and *R. robiginosum* UT/N2 have been growing in axenic conditions in artificial seawater medium (3), since approximately 2005, when they were transferred to the collection of Prof. Andreas Kappler after isolation by Straub et al. (3). To maintain metabolic flexibility, the culture medium was alternatively supplemented with varying substrates (10 mM FeCl_2_, H_2_ (H_2_:CO_2_ 90:10), or 5 mM acetate).

To confirm strain identity, 16S rRNA sequencing was performed for *R. iodosum* UT/N1 and *R. robiginosum* UT/N2 prior to genome sequencing. 1.8 mL of each culture was collected anoxically, and DNA was extracted using the UltraClean R Microbial DNA isolation kit (MO BIO Laboratories, Carlsbad, CS, USA) and then quantified with a Nanodrop ND-1000 Spectrometer (Thermo Scientific, Waltham, MA, USA). The PCR mix consisted of 5x Go Taq reaction buffer (5 µL/sample), MgCl_2_ (1 µL/sample), dNTP Mix (NEB) (0.5 µL/sample), primer forward/reverse (0.5 µL/sample each), Go Taq Polymerase (Promega) (0.125 µL/sample), and water (16.375 µL/sample). Primers 341F (CCTACGGGAGGCAGCAG) and 797R (GGACTACCAGGGTATCTAATCCTGTT) were used (9). The PCR program involved 4 minutes at 96°C, 30 cycles of 1 minute at 96°C, 1 minute at 55°C, and 1 minute at 74°C. This was followed by 10 minutes at 74°C and finally cooling at 4°C.

For genome sequencing, *R. iodosum* UT/N1 DNA extraction was performed using a Qiagen DNeasy Ultraclean Microbial extraction kit and stored at −20°C before sequencing. Sequencing was performed at the Centre for Genomic Research of the University of Liverpool (https://www.liverpool.ac.uk/genomic-research/). An Illumina fragment library was prepared using the NEBNext Ultra II FS kit, targeting ~350 bp inserts. Paired-end sequencing (2 × 150 bp) was done on an Illumina NovaSeq platform using SP chemistry. Illumina adapters were removed using Cutadapt v1.2.1 (10) with the option -O 3, and reads were further trimmed using Sickle v1.2 (11) with a minimum window quality score of 20, removing reads shorter than 15 bp.

Trimmed read files, containing a total of 21,039,110 reads (3.0 Gbp), were analyzed using FastQC v0.11.9 (12). The genome was assembled using SPAdes v3.15.2 in careful mode, with a read coverage cutoff value of 20 and k-mer sizes 21, 33, 55, 61, 71, 81, 91, 101, 111, 121, and 127. The assembly was visualized with Bandage v0.8.1 (13) and extended with SSPACE Standard v3.0 (14). Reads were aligned to the genome using Bowtie v2.4.5 (15), and the genome was submitted to JGI IMG/ER (16) for annotation. Sequence alignments were performed with MAFFT v7.511 (17).

The assembled *R. iodosum* UT/N1 draft genome consists of 11 scaffolds, totaling 3.7 Mbp (N50: 1002450, L50: 2, auN: 1336802) with a GC content of 67.5% (QUAST v5.2.0 [18]), 3,701 annotated genes, and a single 16S rRNA sequence located >800,000 bp from the closest contig boundary (rnammer v1.2 [19]). The genome is estimated to be 99.1% complete (BUSCO v5.4.3 with the rhodobacterales_odb10 data set [20]) with 0.45% contamination (CheckM v1.2.2 [21]) and a mean depth of coverage of 766× (Samtools v1.13-30-ga78376c [22]).

To confirm the identity of the newly sequenced genome, we performed comparisons between the 16S sequence retrieved from our genome (JBEHHI010000001.1 [reverse complement 1052006-1050552]), the sequences deposited by Straub et al. (3) [Y15011.1, Y15012.1], sequences obtained from strains UT/N1 and UT/N2 by Sanger sequencing [PP874721.1, PP874722.1], and the previously sequenced *R. robiginosum* DSM 12329 genome (8) [GCF_003944755.1], which contains a single 16S sequence (NZ_RWGU01000048.1 [222988-224442]) located >140,000 bp from the closest contig boundary (rnammer v1.2). The comparisons (Fig. 1b) confirm that our genome is that of the *R. iodosum* described by Straub et al. in 1999. However, these results also suggest a high degree of similarity between *R. iodosum* UT/N1 and *R. robiginosum* DSM 12329, also confirmed by whole-genome comparisons (99.97% orthoANI with OrthoAniU v1.2 [23], 0 distance with Mash v2.3 [24]). This was unexpected as *R. robiginosum* DSM 12329 is supposed to descend from strain N2 rather than from strain N1. A discrepancy in the 16S sequences is also noted by the DSMZ in the webpages describing the strains.

To further explore this inconsistency, we used blastn v2.13.0+ (25) to search both genomes for the *pufC* gene DNA sequence, which has been previously sequenced from both strains (7). The sequence supposedly from *R. robiginosum* DSM 12329 [AB088690.1] was found in both genomes. However, the “*R. iodosum* DSM 12328” sequence [AB088689.1] was not found in either, suggesting a possible strain swap as early as 2002 (Fig. 1a). Taken together, our findings strongly suggest that our genome represents the *R. iodosum* descended from the original N1 strain and maintained in Tübingen, while the published *R. robiginosum* DSM 12329 genome is more likely that of the strain originally deposited as *R. iodosum* DSM 12328.

Further investigation, including re-sequencing of the DSMZ cultures, is necessary to determine the source of the swap, and previous work should be re-evaluated. As well as providing a new genome for *R. iodosum* UT/N1, this work highlights the importance of confirming taxonomic identity, even for samples obtained from culture collections, and of performing quality control, by comparing new genomes with available sequence data.

## Data Availability

The JGI GOLD analysis project for the *R. iodosum* UT/N1 genome is available with accession number Ga0609869. The genome is accessible from JGI IMG/M with accession number 8025897394 and from GenBank with accession number GCA_041020385.1. Read files as provided by the sequencing centre have been deposited in the Sequence Read Archive with accession number PRJNA1120658.
